# Windblown Lead Carbonate as the Main Source of Lead in Blood of Children from a Seaside Community: An Example of Local Birds as “Canaries in the Mine”

**DOI:** 10.1289/ehp.11577

**Published:** 2008-10-23

**Authors:** Brian Gulson, Michael Korsch, Martin Matisons, Charles Douglas, Lindsay Gillam, Virginia McLaughlin

**Affiliations:** 1 Graduate School of the Environment, Macquarie University, Sydney, New South Wales, Australia;; 2 Exploration and Mining and; 3 Petroleum Research, Commonwealth Scientific and Industrial Research Organisation (CSIRO), North Ryde, New South Wales, Australia;; 4 Department of Health, Perth Business Centre, Western Australia

**Keywords:** adults, blood lead, children, lead carbonate, lead isotopes

## Abstract

**Background:**

In late 2006, the seaside community in Esperance, Western Australia, was alerted to thousands of native bird species dying. The source of the lead was thought to derive from the handling of Pb carbonate concentrate from the Magellan mine through the port of Esperance, begun in July 2005. Concern was expressed for the impact of this process on the community.

**Objective:**

This study was designed to evaluate the source of Pb in blood of a random sample of the community using Pb isotope ratios.

**Methods:**

The cohort comprised 49 children (48 < 5 years of age) along with 18 adults (> 20 years of age) with a bias toward higher blood lead (PbB) values to facilitate source identification.

**Results:**

Mean PbB level of the children was 7.5 μg/dL (range, 1.5–25.7 μg/dL; *n* = 49; geometric mean, 6.6 μg/dL), with four children whose PbB was > 12 μg/dL. The isotopic data for blood samples lay around two distinct arrays. The blood of all children analyzed for Pb isotopes contained a contribution of Pb from the Magellan mine, which for young children ranged from 27% up to 93% (mean, 64%; median, 71%). Subtraction of the ore component gave a mean background PbB of 2.3 μg/dL. Several children whose PbB was > 9 μg/dL and most of the older subjects have complex sources of Pb.

**Conclusions:**

The death of the birds acted as a sentinel event; otherwise, the exposure of the community, arising from such a toxic form of Pb, could have been tragic. Isotopic data and mineralogic and particle size analyses indicate that, apart from the recognized pathway of Pb exposure by hand-to-mouth activity in children, the inhalation pathway could have been a significant contributor to PbB for some of the very young children and in some parents.

In late 2006, the community of about 14,000 people in the town of Esperance in Western Australia (Figure 1) was alerted to several native bird species dying (anecdotes of “birds dropping from the sky”); the number of dead birds exceeded 9,000 (Western Australia Government Committee of Inquiry Education and Health Standing Committee 2007). Early investigations by the Western Australia Department of Agriculture and Food discounted such causes as bird flu and pesticides, and eventually they discovered that the birds were probably dying from lead poisoning. The diagnosis of Pb poisoning was based on significant amounts of Pb residue found in livers, kidneys, and bone and the presence of characteristic Pb inclusion bodies in renal tubular epithelial cells in some birds. Residue levels in livers of birds were found up to 98 mg/kg wet weight (Western Australia Department of Agriculture and Food 2007).

The source of the Pb ore concentrate was thought to derive from the handling of Pb concentrate at the port that began in July 2005. The Pb concentrate originated at the Magellan mine some 600 km north of Esperance and was transported by road and rail to the port (Figure 1). Other products being shipped from the port include grain and nickel and iron ore, but there were no shipments of Pb before those from the Magellan mine. Esperance is located on the coast and faces the Great Southern Ocean and thus experiences strong winds for much of the year. The port is situated close to the center of the town.

After the birds began dying, state and local government authorities began several investigations, although shipping of the ore concentrate continued for several months. Rainwater from household tanks is one of the main sources of drinking water in Esperance. The local Shire Council and Western Australia Department of Health (DoH) undertook measurements of Pb and Ni in the rainwater tanks and initially found that 10% of residences had Pb levels above the World Health Organization guidelines of 10 μg/L and 30% had Ni levels above the guidelines (20 μg/L). The DoH then undertook a blood Pb (PbB) survey of any resident who wished to be tested, and the Department of Environment and Conservation (DEC) undertook environmental investigations. After results from these investigations were obtained, shipping of the concentrate through the port was suspended.

The Pb concentrate being shipped through the port was Pb carbonate, which is an unusual mining product; the most common Pb ore is galena [lead sulfide (PbS)], from which Pb carbonate is derived by oxidation over geologic time. Unfortunately, Pb carbonate is more toxic than PbS: A study by Barltrop and Meek (1975) in rats showed that, compared with an absorption value of 1.0 for Pb acetate, PbS has a value of 0.65, whereas Pb carbonate has a value of 1.69.

When the first author heard about the problem in Esperance, he alerted the DoH and DEC to the highly unusual Pb isotope signature in the ore concentrate, measured before mining by colleagues in the Commonwealth Scientific and Industrial Organisation (CSIRO), and suggested that the Pb isotope method would be a powerful tool in source apportionment investigations. Some questions that could be, and were, addressed using the Pb isotope method were as follows: Is the Pb in the bird livers derived from the Magellan concentrate? If so, what is the pathway from the concentrate to the birds, especially because they were dominantly nectar feeders? Was the Pb adsorbed on the vegetation? Are the elevated Pb levels in rainwater derived from the Magellan concentrate? What are the sources of Pb in soil in parks and residences? What is the contribution of Magellan Pb to PbB of children and adults in Esperance? In this article, we report the results of the isotopic investigation of blood in children and adults. Because the environmental investigations are the subject of litigation, they will be reported at a later date, although the Western Australia Government Committee of Inquiry Education and Health Standing Committee (2007) has stated: “On 4 April 2007, it was confirmed by isotope testing of samples taken from dead birds, soil, water and sediment in Esperance that these matched the lead in samples of Magellan Metals Pty Ltd concentrate transported into the town by rail for export through the Esperance Port” (p. xxiii).

## Materials and Methods

### Subjects and blood sampling

The DoH undertook opportunistic sampling, taking blood from any resident who wished to have his or her PbB measured. All subjects lived within 3 km of the port. None of the subjects were occupationally exposed to Pb, although some of the adults may have undertaken activities in which they were exposed to Pb, such as renovating older houses with Pb paint or making fishing sinkers (Esperance is a seaside town). DoH took most of the samples in a clinic especially set up for the purpose, following standard protocols; a few samples were taken by general medical practitioners in the town. They collected the samples mostly in March through May 2007, after loading of the Pb concentrate had been suspended. Most were venous samples taken with Vacutainer devices, but sometimes by finger prick or heel prick if the parent expressed a preference or they could not obtain a venous sample. Samples we analyzed for Pb isotope ratios were generally for subjects with PbB values > 5 μg/dL, because other studies have pointed out difficulties in assigning sources when PbB values were < 5 μg/dL (Gulson et al. 1996; Manton et al. 2003). For example, Manton et al. (2003) suggest that the success of source apportionment studies using Pb isotopes in humans in the United States is about 1 in 5. We offered children whose PbB values were > 5 μg/dL follow-up with repeat sampling every 3 months until two successive decreases had been recorded. The participants (or the parents/guardians in the case of children) gave written or verbal informed consent before the study began.

### Pb isotope method

**Figure 2:**
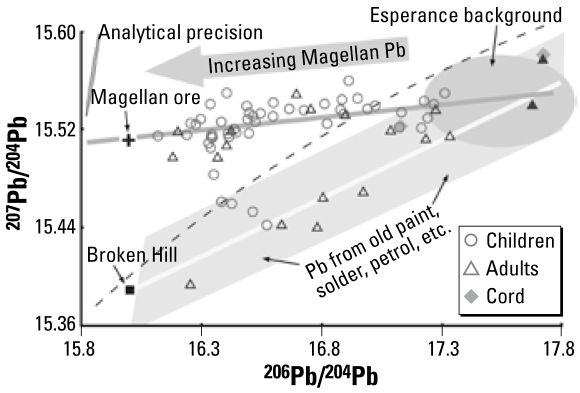
Conventional isotope ratio plot for U-derived Pb isotopes. The dashed line is the growth curve or Pb evolution curve, along which most massive sulfide deposits lie, such as Broken Hill (solid square). The line of best fit for the “Magellan array” isotope data is shown in gray and for the “older array” in white. The shaded fields denoted by arrows are estimations of the possible isotopic fingerprints. The data represented by solid triangles, with the highest ^206^Pb/^204^Pb values, could lie on either array. The 95% confidence ellipse in the upper left corner represents the measuring precision.

The Pb isotope method makes use of the variations, arising from radioactive decay throughout geologic time, in abundances of three of four Pb isotopes, the relative concentrations of Pb, thorium, and uranium, and the time when the ore was formed. Pb has four naturally occurring isotopes, three of which are the stable end products of radioactive decay of U and Th. For example, ^206^Pb is derived by radioactive decay from ^238^U, with a half-life of about 4,500 million years; the decay of ^207^Pb from ^235^U is more rapid, with a half-life of about 700 million years, whereas the decay of ^208^Pb from ^232^Th is much slower, at about 14,000 million years. The other low-abundance isotope, ^204^Pb (~ 1%), has no known radioactive parent and is thought to represent Pb present at the time of formation of the earth, some 4,550 million years ago (so-called primordial Pb); it is used as a reference isotope. Hence, Pb mineral accumulations (mines, deposits) of different geologic ages have different isotopic abundances. The simplest explanation for the different abundances is that when the deposit formed, the Pb was separated from its parent Th and U isotopes, so no further radioactive decay or changes in the isotope abundances occurred. In this way, ore formed, say, 1,800 million years ago had more primordial Pb and relatively fewer decay products compared with ores formed only 400 million years ago; this also includes Pb formed in the intervening 1,400 million years. Isotopic investigations present data as ratios of abundance of one isotope to the other, such as ^208^Pb/^204^Pb, ^207^Pb/^204^Pb, and ^206^Pb/^204^Pb, or any combination of these. In the earlier days of use of Pb isotopes, because of the difficulty in measurement of the low-abundance ^204^Pb isotope, studies reported data as ^206^Pb/^207^Pb ratios. As an example of the differences in isotopes from different ore deposits, the geologically ancient so-called massive sulfide Pb–zinc–silver Broken Hill and Mt. Isa deposits in Australia formed about 1,700–1,800 million years ago and have a ^206^Pb/^204^Pb ratio of 16.0 or 16.1, respectively, whereas geologically younger deposits of similar composition in eastern Australia that formed 400–500 million years ago have a ^206^Pb/^204^Pb ratio of about 18.1. Figure 2 shows the value for the Broken Hill deposits (solid square). The Pb isotope data for most massive sulfide deposits have been found to lie on a so-called Pb evolution or growth curve (the dashed line in Figure 2), which represents the changes in isotope ratios over geologic time (e.g., Gulson 1986).

However, other types of Pb deposits formed by a different mechanism, called Mississippi Valley deposits (found in the Tri-State District of the United States) or sandstone-type deposits (found, e.g., at Pernatty Lagoon in South Australia and in New Mexico and Sweden). Their isotope ratios are very different from those for the massive sulfide deposits, and their data plot away from the growth curves. The Magellan mine is an example of the sandstone type and is thought to have formed about 1,650 million years ago (McQuitty and Pascoe 1998). To account for the unusual isotope ratios in these types of deposits, the Pb is thought to have been sourced from even more ancient, deeply buried rocks, perhaps originally of granitic material (Carr G, Dean J, unpublished data, 1992); hence, the Magellan ore is formed by a multistage process.

### Analytical methods

Thermal ionization mass spectrometry requires good separation of Pb from other elements that may inhibit the ionization of Pb in the mass spectrometer and to avoid interference from ions with the same mass/charge ratio as some of the Pb isotopes. Although Pb levels in the environment have decreased since Pb was removed from gasoline, the processing blank is still a major concern in the design of chemical separation procedures. All reagents we used were ultra-pure, and containers were either Teflon or polypropylene. Under best conditions, we did all processing in class 350 clean rooms with further handling in class 100 laminar flow workbench stations.

Approximately 0.3–0.5 g of blood was digested with concentrated nitric acid/hydrogen peroxide and the Pb separated from other elements on a column of Pb-selective resin (Gale 1996). We added a small amount of ^202^Pb-enriched tracer to determine the Pb concentration in the sample (the isotope dilution method). The procedural blank is about 50 pg (50 × 10^−12^ g), which has an insignificant effect on the measured ratios.

We dissolved the sample in water and evaporated a small volume onto a cleaned rhenium filament along with silica gel/phosphoric acid to enhance ionization. We placed the filaments in a VG Sector 354 multicollector thermal ionization mass spectrometer (VG Isotopes, Norwich, UK) and measured 250 Pb isotope ratios. From replicates of the U.S. National Institute of Standards and Technology (NIST; Gaithersburg, MD, USA) standard reference material SRM 981 and natural samples, a precision of better than ± 0.05% (2σ) for the ^206^Pb/^204^Pb ratios can be obtained, and much better precision can be obtained for the more abundant ratios ^208^Pb/^206^Pb and ^207^Pb/^206^Pb. To enable comparisons of data across laboratories, we normalized the isotope ratios to the accepted values of the NIST standard SRM 981.

## Results and Discussion

**Figure 3:**
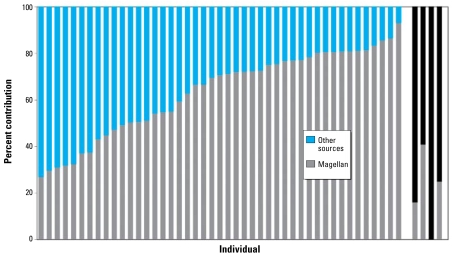
Percentage of Magellan Pb in Esperance children ≤ 6 years of age. The black bars are for children whose data lie toward the array line in Figure 2.

**Figure 4:**
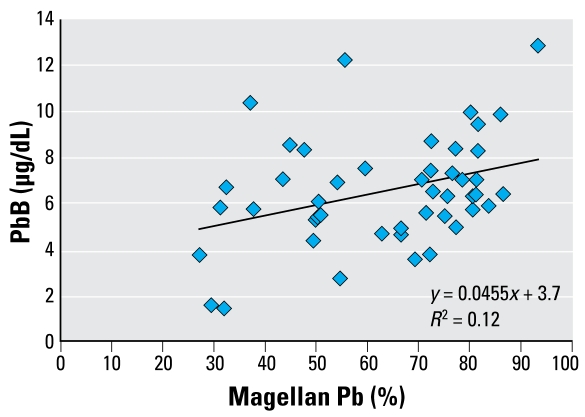
Percentage of Magellan Pb versus PbB concentration in young children from Esperance. The data for four children (W610, W711, W712, W716) are omitted because the Magellan concentrate was not the dominant source of Pb in their blood.

We present our results in Figures 2–4 and Table 1. We examined Pb isotopes in blood from 48 children < 5 years of age, one 6 years of age, 18 adults > 20 years of age, and two cord blood samples.

Isotope data may be presented in a variety of ways, but the ^207^Pb/^204^Pb versus ^206^Pb/^204^Pb graph (Figure 2) is the most illustrative for the present study. The 95% confidence ellipse in the upper left corner illustrates a conservative estimate of the analytical precision of the isotope measurements, and the dashed line is the growth curve or Pb evolution curve described above.

### Signature for the ore

The isotopic signature of the Magellan ore is shown by the black cross on the left side of Figure 2. This signature is highly distinctive, lying well above the growth curve. A search of the extensive unpublished CSIRO database (containing > 3,000 groups of Pb isotope data) for other Australian deposits shows no comparable signature for any prospect/deposit currently being mined. This is an unusual signature in the Australian environment. In the past, we have often illustrated the Pb isotope results using, for example, only the ratio ^206^Pb/^204^Pb versus time. In the present case, use of this ratio alone would not have been discriminatory because several geologically ancient Pb deposits, such as Broken Hill in New South Wales (NSW), and Mt. Isa and Cannington in Queensland, have ^206^Pb/^204^Pb ratios almost identical with Magellan because they formed at much the same geologic time. However, these other deposits have ^207^Pb/^204^Pb and ^208^Pb/^204^Pb ratios different from Magellan’s, because the U, Th, and Pb were partially mobilized at Magellan before the ore body was formed. Using the ratios ^207^Pb/^204^Pb versus ^206^Pb/^204^Pb on a graph allows us to discriminate Magellan deposits from these other deposits, and this combination provides better visual resolution than does a graph of ^208^Pb/^204^Pb versus ^206^Pb/^204^Pb or even of the more abundant ratios ^208^Pb/^206^Pb versus ^207^Pb/^206^Pb.

### Estimation of contributions of Magellan Pb in blood

It is possible to calculate the contributions of PbB from different sources using well-established methods from isotope geochemistry assuming two-component mixing (Faure 1986). More complex mixing relationships are possible and could explain some of the results, especially for the adults and the children with the highest PbB levels. Any point on a graph of isotope ratios can represent a single homogeneous source or can be a mix of two or more isotopically different sources. When Pb from two sources is mixed, the resultant isotope value will lie on a straight line between the end points. The distance along that line is proportional to the ratio of the amounts of Pb from each of the sources. Thus, a 50:50 mix will give a point halfway along that line.

The isotopic data confirm the presence of two quite distinct linear arrays, defined by *a*) most of the PbB data that intersects the Magellan ore value and includes the subjects with the lowest PbB of < 1.5 μg/dL (“Magellan array”; gray line in Figure 2) and *b*) another array (“older array”; white line in Figure 2) around which lie most of the isotope data for older subjects (> 20 years of age; triangles in Figure 2) and three water samples (data not shown). The white line is the best fit through the isotopic data for the geologically ancient Broken Hill deposit in NSW and younger Mississippi Valley deposits from the United States. We have observed mixing lines of similar slope in several other investigations, and, in fact, a line of best fit through the blood isotopic data for > 300 Australian residents from differing Pb exposure, as well as > 100 migrant subjects to Australia, has a slope similar to the white line in Figure 2.

For the estimations of the contribution to PbB, we have used the isotopic values for Magellan concentrate as one end point. The isotopic value for the other (“low-Magellan”) end point is uncertain because no attempt so far has been made to identify and isotopically characterize other Pb sources such as paint, hobbies, and auto repairs in Esperance. The low-Magellan end point has been arbitrarily assigned a value of 17.8 for the ^206^Pb/^204^Pb ratio. An alternative end point value could be 17.3, which is the isotope ratio for two subjects with PbB < 2 μg/dL (the left end of the Esperance ellipse in Figure 2). Using such a value would decrease the contributions attributed to the Magellan end point by about one-quarter. However, we preferred the higher value because it is based on the intersection of the Magellan and older arrays and on data from a cord blood, and two adult blood samples, and water samples.

### Children ≤ 6 years of age

The mean PbB level of the children for whom Pb isotope compositions were available was 7.5 μg/dL, with a range of 1.5–25.7 μg/dL (*n* = 49) and geometric mean of 6.6 μg/dL. This compares with the mean value of 3.0 μg/dL obtained for 404 children < 5 years of age in the wider DoH survey (Western Australia DoH 2007a). However, we biased the cohort for the Pb isotope sampling toward higher PbB values to evaluate the sources of Pb.

Assuming that the low-Magellan end point value is valid, it would appear that the PbB of all the children analyzed for Pb isotopes contains a proportion of Magellan Pb. The contribution of Magellan Pb to PbB in young children ranged from 27% up to 93% in one child only 6 months of age, with a mean of 64% and median of 71%.

The isotopic data for three children lie between the Magellan and older arrays. PbB levels in these three children ranged from 12 to 25 μg/dL, which suggests that there is another source (or sources) of Pb that has an isotopic composition probably lying on the older array with a ^206^Pb/^204^Pb value of about 16.6. For these children, one end point could be Magellan Pb and the other a mixture of “normal”-type Pb such as from the Broken Hill mines and a geologically younger Pb. With such end points, the Magellan contribution would be about 10–50%. However, with so few data points, such calculations are uncertain. The non-Magellan source could be paint, car batteries, ingestion of soil containing Pb from fuel/engine oil (if maintenance was carried out in the yard), or myriad others. Unfortunately, no environmental samples from individual houses were available for isotopic analysis.

Figure 3 shows the relative Pb contribution to PbB in children < 6 years of age from Magellan ore. A plot of PbB versus percent Magellan Pb in blood of the children shows a significant positive relationship (*R*^2^ = 0.12, *p* = 0.018) of increasing PbB and proportion of Magellan Pb (Figure 4). Data for children whose Pb isotope data indicate a major source of Pb other than from Magellan ore (W610, W711, W712, W716) do not appear in Figure 4.

Several children with elevated PbB had follow-up samplings about 3 months after the initial sampling. All except one had reduced PbB levels since the cessation of Pb exports, combined with cleaning of houses and other interventions. We hoped that isotopic analysis may help identify possible other sources of Pb in these children’s environments that had been overlooked. However, because no environmental samples from individual houses were available for analysis, such identification could only be surmised.

### Adults

The data for the 18 adults > 20 years of age lie on both arrays, with the data for 7 subjects lying on the lower section of the older array, 10 subjects lying on the Magellan array, and 2 subjects that could lie on either array (solid triangles in Figure 2).

The data for seven of the subjects whose PbB ranged from 7.3 to 20.3 μg/dL lie on the older array, along with three samples of tank rainwater collected from a suburb approximately 2 km from the port (data not shown). The data for two subjects, however, could possibly lie on the Magellan array. The isotopic compositions suggest that another source(s) of Pb is present, such as Pb from maternal bone, paint, roof flashing, tank water, hobbies, do-it-yourself activities, unusual diet, medicines, cosmetics, or occupational activities. No environmental samples from individual houses were available for isotopic analysis.

The data for 10 subjects, whose PbB ranged from 1.8 to 14.2 μg/dL, lie on the Magellan array and indicate that in some cases they have been exposed to Magellan Pb; the contribution of Magellan Pb to PbB ranged from 29% to 77%. The data, however, for two of these subjects, with low Magellan contributions of 29% and 40%, lie equally well on the older array, along with the data for subjects W725 and W747.

The data for the cord blood samples are consistent with those found in other studies; that is, the isotope ratios in the cord and mothers’ blood are similar (Gulson et al. 1997, 1998, 2004a; Manton et al. 2003). Despite the higher PbB concentrations in the mothers compared with their offspring, the low concentrations in the cord blood suggest an encouragingly low transfer of Pb to the child. One cord blood sample with a PbB of about 2 μg/dL (W746, mother W747 in Table 1) had a contribution of Magellan Pb of about 37%. The other cord blood sample, with < 1 μg/dL PbB, had an insignificant amount of Magellan Pb (W748, mother W749).

### Estimated background PbB values for young children

It is possible to estimate a background PbB value expected in Esperance if there had been no significant Pb exposure from a Magellan source. Excluding the four children (W610, W711, W712, W716 in Table 1) whose Pb isotope ratios indicate mostly a non-Magellan source, subtraction of the mean contribution from Magellan ore gives residual PbB values between about 1 and 4 μg/dL. The geometric mean background PbB is about 2.3 μg/dL. An alternative method to estimate the background PbB derives from the slope in Figure 4. For 0% Magellan Pb, the extrapolated value is about 3.7 μg/dL, which is higher than the above value, but at this stage we cannot explain the difference.

Values at these levels or even lower would be expected for Esperance. We have measured a geometric mean PbB value of 2.6 μg/dL in a cohort of 114 Sydney children, most of whom have been monitored every 6 months for more than 5 years (Gulson et al. 2006). Given the Pb contamination in Sydney over several decades, especially from gasoline, low PbB values would be expected for a remote community such as Esperance. For comparison, the U.S. National Health and Nutrition Examination Survey 1999–2002 data for children 1–5 years of age, for all racial/ethnic groups, is 1.6 μg/dL (Centers for Disease Control and Prevention 2005).

### Some thoughts on pathways

It is well recognized nowadays that the most common pathway for Pb exposure in young children is by hand-to-mouth activity. In Esperance, the fine particle size of the ore concentrate (discussed below) and strong ubiquitous winds have resulted in widespread contamination, although only limited environmental sampling of houses has been undertaken. Limited soil sampling around residential parks and school playgrounds undertaken by the Western Australia DEC showed a highest reading of 88 mg/kg, and most were < 10 mg/kg (Western AustraliaDoH 2007b). The DoH took dust wipe samples from households where residents had elevated PbB levels (either adults with PbB levels > 10 μg/dL or children < 5 years of age with PbB levels ≥ 5 μg/dL). Twenty-one dust wipes were taken inside homes from a variety of surfaces, including cupboards, shelves, appliances, windows, venetian blinds, and door frames. Pb content of wipes ranged from 0.014 to 1.1 μg/cm^2^ (mean, 0.21 μg/cm^2^). Twenty-two wipe samples were taken from a variety of locations on or outside dwellings, including windows, doors, beams, cubbyhouses, and other outdoor structures. Pb content in these wipes ranged from 0.16 to 34 μg/cm^2^ (mean, 4.86 μg/cm^2^). There are no guidelines for dust wipes apart from those promulgated by U.S. Department of Housing and Urban Development (HUD) (Clickner et al. 2001) for cleanup after Pb-based paint remediation. These values are 0.043 μg/cm^2^ for floors and 0.27 μg/cm^2^ for windowsills.b The Western Australia DoH (2007b) recommended that Pb in dust on surfaces should be 0.04 μg/cm^2^ or lower in areas accessible to young children and 0.4 μg/cm^2^ for other surfaces around the home. Many of the Esperance samples thus exceed the HUD and DoH guidelines.

The high percentage of Magellan Pb in one 6-month-old child (93%) and in both parents (~ 76%) would suggest that the inhalation pathway of Pb may have been a significant contributor to the overall PbB level. Furthermore, per unit body weight, ventilation rates are much higher in infants than among children or adults. The family lived within 200 m and downwind of the Pb loading shed. The child was not crawling and exhibited limited if any hand-to-mouth activity, according to the parents. The inhalation pathway is unusual these days, apart from occupational exposure, in contrast to the 1970s and 1980s when leaded gasoline was in use. The hypothesis of an inhalation pathway to complement hand-to-mouth activity in many of the children is reinforced by the nature of the Magellan ore concentrate and the high levels of Pb in air measured by the Port Authority using deposition gauges, which measure the particle deposition from the air over a 30-day period. These gauges showed very high levels of Pb, especially during periods of loading the concentrate onto ships. Values ranging from 14 to 42 mg/m^2^/month were reported from August 2005 to August 2006 (Sinclair Knight Mertz 2007). No guidelines are available for deposition gauge measurements, but the results compare with low values we have measured using petri dish collection in Sydney during 2001–2006 of 0.03 mg Pb/m^2^/30 days (geometric mean).

The Pb concentrate from the Magellan mine was originally supposed to be transported by rail as pellets or agglomerates, but the company argued against this because of problems with handling on the wharf (Western Australia Government Committee of Inquiry Education and Health Standing Committee 2007). We subjected a sample of concentrate from the port to mineral identification using X-ray diffraction as well as microscopic and laser particle size analysis. The X-ray analysis showed the concentrate to consist of approximately 76% Pb carbonate, about 5% PbS (galena), and the rest Pb sulfate. Pb carbonate is recognized as having a much higher absorption than the other compounds, especially PbS (Barltrop and Meek 1975). The laser particle size analysis showed that more than 40% of the particles were < 10 μm and 13% < 2.5 μm in diameter, and microscopic examination confirmed an abundance of fine particles < 10 μm in size. Such particles can penetrate into the alveolar region of the lungs, where the rate of absorption of Pb particles is very high [32% deposition in the lungs, where it is mostly absorbed—the default value in the U.S. Environmental Protection Agency’s integrated exposure uptake biokinetic model for children (U.S. Environmental Protection Agency 1994)].

### Comparison with other communities

Elevated PbB levels are commonly found in mining and smelting communities, although those producing Pb carbonate and/or subject to transportation are less common and isotopic studies even more so. One example where Pb carbonate was produced is in the Pb–zinc–silver mines of Broken Hill, NSW, Australia. Mining began in the 1880s with open pit methods, initially of carbonate ore, which later changed to underground mining of sulfide ore. An inquiry into the prevalence of Pb poisoning and deaths among the Broken Hill community was reported in 1893 (NSW Government Printer 1893). A PbB prevalence survey undertaken by NSW Health in 1991 showed that > 85% of children < 5 years of age had PbB levels > 10 μg/dL (Lyle et al. 2006). Such high PbB levels were attributed by Gulson et al. (1994a) to the oxidized nature of the highly soluble Pb species, in contrast to many other mining communities where the Pb minerals were either galena (PbS) or encapsulated in relatively insoluble minerals (Bornschein et al. 1991; Cotter-Howells and Thornton 1991; Ruby et al. 1992; Steele et al. 1990). A major Pb isotope investigation involving 366 samples was undertaken to determine the source and pathways of Pb in the Broken Hill mining community (Gulson et al. 1994b, 1996). Blood, urine, and environmental samples were obtained from 27 families encompassing 60 children 1–11 years of age, 41 female adults, and 15 male adults. Environmental samples included soils, gutter sweepings, ceiling (attic) dust, vacuum cleaner dust, long-term dust, surface dust wipes, external and internal air, food, water, and gasoline. Potential sources of Pb were from the Pb–zinc–silver ore body, from paint, and from gasoline. Thirty-five of the 60 children (60%) had PbB levels > 15 μg/dL, compared with a “background” level of approximately 6 μg/dL estimated from adult females who were generally mothers of the children. Six of 17 children ≥ 7 years of age had PbB > 15 μg/dL. Even though the ore-body Pb was the major contributor to PbB in Broken Hill children, of the 35 children whose PbB was > 15 μg/dL, for 12 (34%) approximately 50% or more of their PbB derived from sources such as paint and gasoline or both, based on isotopic identification.

In an example of “take-home” Pb dust, Pb isotope and concentration data were obtained from venous blood and environmental samples (vacuum cleaner dust, interior dustfall accumulation, water, paint) for eight children of six adult male employees and the adults who worked in a Pb–zinc–copper mine located approximately 40 km from the town (Chiaradia et al. 1997). These data were compared with results for 11 children from nonoccupationally exposed (“control”) families living in the same town. Pb in blood of the mine employees varied from 7 to 25 μg/dL and was generally dominated by mine PB (> 60%). The mean PbB (± SD) in the children of the mine employees was 5.7 ± 1.7 μg/dL, compared with 4.1 ± 1.4 μg/dL for the control children (*p* = 0.02). Some of the “control” children had higher PbB than the children of mine employees, probably from exposure to leaded paint, because six of eight houses for the control children were > 50 years of age. Five of eight children of mine employees had PbB contributions from mine Pb > 20%. However, the other three children of mine employees had contributions to their PbB from sources other than mine Pb (possibly paint, gasoline, background sources). This study showed that houses of employees from a Pb mine can be variably contaminated by mine Pb even if they are not near the mine.

Gulson et al. (2004b) compared high-precision Pb isotopic ratios in sectioned deciduous teeth and environmental samples to evaluate sources of Pb in 10 children from six houses in a primary zinc–Pb smelter community at North Lake Macquarie, NSW, Australia. PbB levels in the children ranged from 10 to 42 μg/dL and remained elevated for a number of years. The median Pb level in the enamel section of the teeth was 2.3 μg/g, with a range of 0.6–7.4 μg/g; in dentine, the median was 5.3 μg/g with a range of 1.4–19.9 μg/g. For most children, only a small contribution to tooth Pb can be attributed to gasoline and paint sources. Comparison of isotopic ratios of tooth Pb levels with those from vacuum cleaner dust, dustfall accumulation, surface wipes, ceiling (attic) dust, and an estimation of the smelter emissions indicated that from approximately 55–100% of Pb could be derived from the smelter. For a blood sample from another child, we estimated > 90% of Pb to derive from the smelter. In contrast to other studies on teeth from children from other Pb mining and smelting communities, the levels of Pb in the teeth were surprisingly low.

Naeher et al. (2003) measured Pb isotopic ratios in samples of gasoline, air particulates, mineral concentrate, and blood from children in Peru. One group of children (*n* = 20) whose PbB levels were > 19 μg/dL lived in Callao, a port that stored and shipped mineral concentrate; the isotopic results were compared with another group of 10 children from outside Callao whose PbB levels were < 10 μg/dL. The isotopic results for the two groups of children were very different, and both were different from five samples of gasoline; the results for the children from Callao were almost identical to those in the mineral concentrate.

Tsuji et al. (2008) identified Pb ammunition as a source of Pb exposure for First Nations people of Canada by using Pb isotopes. They suggested Pb-contaminated meat from game shot with Pb bullets may also be a contributor to the Pb body burden.

## Conclusions

This incidence of avian Pb poisoning in a pristine environment came as a shock to the community of Esperance. Although the deaths of so many native birds is a tragedy, their role as “canaries in the coal mine” is fortunate for the community, which may otherwise have been left with a legacy so common in many mining and smelting environments. This would have been exacerbated by the Pb concentrate being in the form of Pb carbonate and of such fine particulate size, combined with the ubiquitous strong winds coming off the Great Southern Ocean, resulting in wide dispersion of the material.

The other fortunate aspect of this event is the unusual Pb isotopic signature of the Pb concentrate, which has allowed unequivocal identification of the source and contribution of the Pb concentrate to the blood of children and adults in the community, to the birds, and to the environmental samples. If the signature were less distinct, it would not be possible to apportion sources of the Pb with any certainty where PbB levels are < 5 μg/dL, a potential problem noted previously by Gulson et al. (1996) and Manton et al. (2003). Furthermore, the Pb isotope results allow a background PbB to be estimated.

The major limitation of this investigation is the lack of isotopic data for environmental samples within individual houses, which would have allowed identification of the other sources of Pb. This is especially the case for the children with the highest PbB levels of > 15 μg/dL and whose isotopic results showed that the overwhelming contribution of Pb in their blood was from sources other than the Pb concentrate. Another limitation is the lack of knowledge of potential sources of Pb besides from the concentrate. None of the subjects were occupationally exposed to Pb, although some of the adults may have undertaken activities that exposed them to Pb, such as renovating older houses with Pb paint or making fishing sinkers.

This study has shown that the isotopic analysis of environmental and blood samples can contribute significantly to the investigation of elevated Pb levels. Investigation of such episodes should include the early development of protocols and procedures to enable systematic and valid sampling of environmental and blood specimens for isotopic analysis.

## Figures and Tables

**Figure 1:**
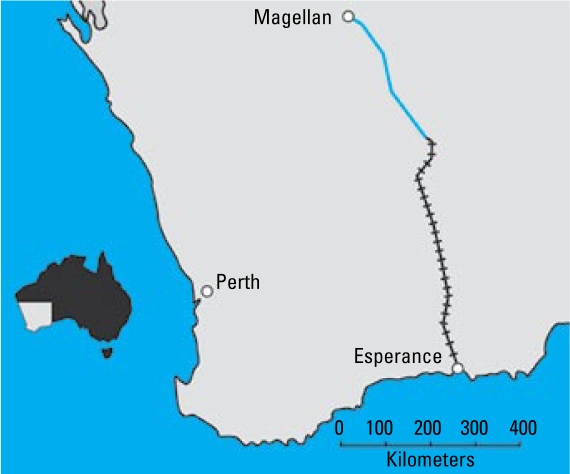
Location of Esperance, Western Australia. The Pb carbonate concentrate is transported by road (blue line) and rail (hatched line) from the Magellan mine to Esperance.

**Table 1:** Pb isotopic ratios and concentration in Esperance subjects.

Lab no.	Age (years)	^208^Pb/^206^Pb	^207^Pb/^206^Pb	^206^Pb/^204^Pb	^207^Pb/^204^Pb	^208^Pb/^204^Pb	PbB (μg/dL)	% Magellan Pb
Children								
W597	0.3	2.1934	0.9494	16.343	15.516	35.845	5.7	81
W602	0.4	2.2007	0.9555	16.238	15.515	35.736	6.5	86
W713	0.5	2.2084	0.9625	16.119	15.514	35.597	12.8	93
W619	0.6	2.1872	0.9425	16.487	15.539	36.062	6.5	73
W610	0.6	2.1980	0.9412	16.426	15.459	36.104	9.1	NA
W738	0.6	2.1552	0.9017	17.213	15.521	37.098	6.8	33
W740	0.6	2.1833	0.9364	16.598	15.541	36.238	5.0	67
W617	0.7	2.1970	0.9469	16.350	15.483	35.922	9.8	80
W718	0.8	2.1660	0.9172	16.948	15.545	36.709	8.4	47
W737	0.8	2.1642	0.9201	16.911	15.559	36.599	4.4	49
W605	0.8	2.1994	0.9529	16.296	15.528	35.842	5.9	83
W608	0.9	2.1691	0.9246	16.805	15.538	36.453	12.2	55
W608	0.9	2.1979	0.9538	16.276	15.524	35.773	8.0	84
W712	1.0	2.1986	0.9442	16.391	15.476	36.038	24.7	NA
W724	1.0	2.1899	0.9456	16.410	15.516	35.936	8.4	77
W606	1.1	2.1946	0.9495	16.339	15.514	35.858	9.4	81
W731	1.2	2.1953	0.9479	16.405	15.549	36.012	5.0	77
W736	1.3	2.1897	0.9443	16.436	15.520	35.989	6.3	76
W716	1.4	2.1883	0.9319	16.571	15.442	36.263	11.9	NA
W611	1.5	2.1949	0.9498	16.329	15.509	35.840	8.3	81
W717	1.5	2.1787	0.9323	16.664	15.536	36.307	4.7	63
W604	1.5	2.1659	0.9206	16.877	15.536	36.552	5.6	51
W629	1.6	2.1492	0.9024	17.224	15.543	37.017	1.5	32
W739	1.6	2.1442	0.8983	17.310	15.549	37.116	3.8	27
W730	1.7	2.1857	0.9397	16.523	15.526	36.115	7.1	71
W598	1.8	2.1865	0.9405	16.514	15.530	36.106	5.6	71
W723	2.0	2.1663	0.9212	16.885	15.554	36.576	6.0	51
W722	2.2	2.2013	0.9551	16.254	15.525	35.782	9.8	86
W600	2.3	2.1658	0.9196	16.890	15.532	36.581	5.4	50
W735	2.3	2.1939	0.9478	16.382	15.526	35.941	7.0	79
W607	2.3	2.1955	0.9490	16.338	15.505	35.870	6.5	81
W599	2.4	2.1892	0.9437	16.444	15.517	35.999	5.5	75
W711	2.4	2.1916	0.9358	16.513	15.453	36.189	23.5	NA
W734	2.4	2.1766	0.9288	16.726	15.535	36.405	7.5	59
W609	2.5	2.1969	0.9513	16.336	15.540	35.890	7.0	81
W612	2.5	2.1880	0.9416	16.491	15.528	36.083	8.7	72
W733	2.7	2.1700	0.9229	16.821	15.524	36.502	6.9	54
W721	2.8	2.1528	0.9068	17.130	15.534	36.878	10.4	37
W631	3.1	2.1692	0.9244	16.811	15.541	36.466	2.8	55
W719	3.4	2.1638	0.9145	16.989	15.536	36.761	8.5	45
W729	3.4	2.1874	0.9406	16.496	15.517	36.084	7.3	72
W728	3.7	2.1540	0.9066	17.121	15.521	36.878	5.8	38
W603	4.1	2.1823	0.9358	16.597	15.530	36.219	4.8	67
W601	4.1	2.1941	0.9491	16.345	15.513	35.863	6.4	81
W596	4.2	2.1848	0.9388	16.545	15.533	36.146	3.6	69
W732	4.2	2.1897	0.9452	16.413	15.513	35.940	7.3	77
W727	4.4	2.1493	0.9008	17.239	15.529	37.052	5.8	31
W726	4.7	2.1571	0.9129	17.021	15.539	36.716	7.1	43
W630	4.8	2.1462	0.9001	17.265	15.540	37.054	1.6	30
W743	5.9	2.1860	0.9416	16.497	15.533	36.063	3.8	72
Adults								
W720	22.0	2.1422	0.9002	17.231	15.511	36.913	3.4	32
W749	23.9	2.1186	0.8791	17.717	15.576	37.536	1.2	5 or E
W748	Cord	2.1187	0.8793	17.719	15.580	37.541	0.9	5 or E
W742	28.9	2.1754	0.9314	16.694	15.548	36.316	5.3	61
W745	30.5	2.1756	0.9202	16.804	15.464	36.559	18.2	55
W715	33.2	2.1900	0.9449	16.423	15.518	35.965	6.8	76
W725	38.1	2.1407	0.8994	17.272	15.535	36.975	1.8	29
W623	38.8	2.1247	0.8792	17.673	15.539	37.551	20.4	7 or E
W714	41.3	2.1903	0.9453	16.404	15.506	35.931	9.6	77
W741	42.0	2.1732	0.9273	16.753	15.536	36.408	6.2	58
W747	44.0	2.1515	0.9083	17.085	15.518	36.759	2.0	40
W746	Cord	2.1509	0.9063	17.126	15.522	36.837	2.0	37
W616	52.1	2.1804	0.9284	16.633	15.442	36.265	8.3	NA
W622	54.6	2.2041	0.9578	16.201	15.517	35.710	14.2	89
W620	60.4	2.1650	0.9192	16.897	15.531	36.582	11.0	50
W624	66.9	2.2075	0.9471	16.253	15.393	35.878	19.4	NA
W615	67.0	2.2039	0.9578	16.180	15.497	35.659	10.5	90
W613	70.2	2.1363	0.8951	17.331	15.514	37.025	10.2	NA
W621	78.1	2.1721	0.9201	16.780	15.439	36.448	13.4	NA
W614	84.0	2.1605	0.9114	16.971	15.468	36.667	7.3	NA
W618	85.3	2.1918	0.9468	16.367	15.496	35.872	10.8	79

Abbreviations: E, data could lie on either array; NA, not applicable because data lie toward older array.

a Samples W748/W749 and W746/W747 are cord/mother blood.
